# Increase in Mitochondrial Biogenesis in Neuronal Cells by RNS60, a Physically-Modified Saline, via Phosphatidylinositol 3-Kinase-Mediated Upregulation of PGC1α

**DOI:** 10.1007/s11481-017-9771-4

**Published:** 2017-11-29

**Authors:** Goutam Chandra, Madhuchhanda Kundu, Suresh B. Rangasamy, Sridevi Dasarathy, Supurna Ghosh, Richard Watson, Kalipada Pahan

**Affiliations:** 1 Department of Neurological Sciences, Rush University Medical Center, 1735 West Harrison St, Suite Cohn 310, Chicago, IL 60612 USA; 2 Revalesio Corporation, 1200 East D Street, Tacoma, WA 98421 USA

**Keywords:** Mitochondrial biogenesis, Neurons, PGC1α, Modified saline, Nanobubbles, PI3 kinase

## Abstract

This study highlights a novel approach to upregulate mitochondrial biogenesis in neuronal cells. RNS60 is a 0.9% saline solution containing oxygenated nanobubbles that is generated by subjecting normal saline to Taylor-Couette-Poiseuille (TCP) flow under elevated oxygen pressure. RNS60, but not NS (normal saline), PNS60 (saline containing a comparable level of oxygen without the TCP modification), or RNS10.3 (TCP-modified normal saline without excess oxygen), increased the expression of *Nrf1*, *Tfam*, *Mcu*, and *Tom20* (genes associated with mitochondrial biogenesis) and upregulated mitochondrial biogenesis in MN9D dopaminergic neuronal cells. Similarly RNS60 also increased mitochondrial biogenesis in primary dopaminergic neurons and in the nigra of MPTP-intoxicated mice. However, RNS60 had no effect on lysosomal biogenesis. Interestingly, we found that RNS60 upregulated PGC1α and siRNA knockdown of PGC1α abrogated the ability of RNS60 to increase mitochondrial biogenesis. Furthermore, we delineated that RNS60 increased the transcription of *Pgc1a* via type IA phosphatidylinositol (PI) 3-kinase-mediated activation of cAMP-response element-binding protein (CREB). Accordingly, knockdown of the PI3K – CREB pathway suppressed RNS60-mediated mitochondrial biogenesis. These results describe a novel property of RNS60 of enhancing mitochondrial biogenesis via PI 3-kinase-CREB-mediated up-regulation of PGC1α, which may be of therapeutic benefit in different neurodegenerative disorders.

## Introduction

Mitochondrion is the most important organelle in terms of energy metabolism and neurons in the central nervous system (CNS) have an intense demand for mitochondrial function. Recent studies indicate alteration in mitochondrial homeostasis in the CNS of patients with different neurodegenerative diseases. While complex I activity is reduced in I in Parkinson’s disease (PD), activities of cytochrome oxidase and multiple electron transport chain (ETC) enzymes are decreased in Alzheimer’s disease (AD) and Huntington’s disease, respectively (Mizuno et al. 1995; Hirai et al. 2001; Baloyannis 2006; Surmeier and Sulzer 2013; Guedes-Dias et al. 2015). Mitochondrial dysfunction has also been reported in amyotrophic lateral sclerosis and progressive supranuclear palsy (Albers and Beal 2002; Tafuri et al. 2015). Leber’s hereditary optic neuropathy, a focal degeneration of the optic nerves, is known to arise due to mutations in mtDNA-encoded complex I genes and is associated with complex I dysfunction (Carelli et al. 2004). Mitochondrial dysfunction also participates in the pathogenesis of epilepsy (Zsurka and Kunz 2015). Since different neurodegenerative diseases are associated with mitochondrial malfunction, it is important to focus on developing methods to repair and restore mitochondria. Enhancing mitochondrial biogenesis may have therapeutic importance for different neurodegenerative diseases.

The diverse functionality of mitochondria requires a very complex and coordinated regulation of its activity with transcription factors NRFs/PPARs/ERRs that activate target genes encoding enzymes of fatty acid metabolism, oxidative phosphorylation and anti-oxidant defense (Scarpulla 2002; Fan and Evans 2015). By co-activating and regulating the expression of this network, PGC1α, the master regulator of mitochondrial biogenesis, directly links external physiological stimuli to the regulation of mitochondrial biogenesis and function (Wu and Boss 2007; Wareski et al. 2009). However, mechanisms by which PGC1α is activated and mitochondrial biogenesis is upregulated are poorly understood. RNS60 is physically modified saline that contains no active pharmaceutical ingredients. It is generated by subjecting normal saline to Taylor-Couette-Poiseuille (TCP) flow under elevated oxygen pressure (Khasnavis et al. 2012; Mondal et al. 2012; Roy et al. 2014). Recently, we have demonstrated that RNS60 exerts anti-inflammatory effects in glial cells via suppression of nuclear factor kappa B (NF-κB) activation (Khasnavis et al. 2012). RNS60 also protects dopaminergic neurons in MPTP mouse model of PD (Khasnavis et al. 2014) and hippocampal neurons in a mouse model of AD (Modi et al. 2015). Here, we delineate that RNS60 stimulated mitochondrial biogenesis and increased the expression of *Nrf1*, *Tfam*, *Mcu*, and *Tom20* (genes associated with mitochondrial biogenesis) in dopaminergic neuronal cells. However, RNS60 did not have any such stimulatory effect on lysosomal biogenesis. Furthermore, we demonstrate that RNS60 induced the activation of type IA PI3K and that RNS60 increased mitochondrial biogenesis via type IA PI3K-CREB-mediated upregulation of PGC1α. Finally, MPTP intoxication reduced the expression of PGC1α and decreased mitochondrial biogenesis, which were increased by RNS60 treatment. Our studies suggest that this physically-modified saline may be of therapeutic value in the treatment of PD and other neurodegenerative disorders in which mitochondrial abnormality plays an important role.

## Methods

### Cells

MN9D cells were obtained from Dr. A. Heller (University of Chicago, Chicago, IL, USA). Cells were maintained in Dulbecco’s modified Eagle’s medium (Thermo Fisher Scientific, Waltham, MA) supplemented with 10% (*v/v*) heat-inactivated fetal bovine serum, 3.7 g/L NaHCO_3_, 50 U/mL penicillin, and 50 μg/mL streptomycin in an incubator with an atmosphere of 7% CO_2_ at 37°C. These cells express abundant tyrosine hydroxylase, synthesize dopamine (DA) and also quantitatively release DA (Choi et al. 1991; Perez et al. 2002; Dong et al. 2008).

### Reagents

Hank’s balanced salt solution (HBSS) and 0.05% trypsin were purchased from Mediatech (Washington, DC). Fetal bovine serum (FBS) was obtained from Atlas Biologicals (Fort Collins, CO). Antibiotic-antimycotic mixture was obtained from Sigma-Aldrich (St. Louis, MO).

### Isolation of Mouse Primary Dopaminergic Neurons

Nigra was dissected as a thin slice of ventral mesencephalon tissue from E12.5 to 14 days old fetus and homogenized with 1 ml of trypsin for 5 min at 37°C followed by neutralization of trypsin as described earlier (Roy and Pahan 2013). The single cell suspension of nigral tissue was plated in the poly-d-lysine pre-coated 75 mm flask and was allowed to differentiate fully for 9–10 days before treatment (Roy and Pahan 2013).

### Animals and MPTP Intoxication

Six- to eight-weeks old C57BL/6 mice were purchased from Harlan, Indianapolis, IN. Animal maintenance and experiments were in accordance with National Institutes of Health guidelines and were approved by the Institutional Animal Care and Use committee of the Rush University Medical Center, Chicago, IL. For acute MPTP intoxication, mice received four intraperitoneal (i.p.) injections of MPTP-HCl (18 mg/kg of free base; Sigma Chemical Co., St. Louis, MO) in saline at 2-h intervals (Ghosh et al. 2007; Ghosh et al. 2009; Roy et al. 2012). Control animals received only saline.

### Preparation of RNS60

RNS60 was generated at Revalesio (Tacoma, WA) as described before (Khasnavis et al. 2012; Mondal et al. 2012). Briefly, sodium chloride (0.9%) for irrigation, USP pH 5.6 (4.5–7.0, Hospira), was processed at 4°C using Taylor-Couette-Poiseuille (TCP) flow and a flow rate of 32 mL/s under 1 atm of oxygen back-pressure (7.8 mL/s gas flow rate), while maintaining a rotor speed of 3450 rpm. Chemically, RNS60 contains water, sodium chloride, 50–60 ppm oxygen, but no active pharmaceutical ingredients. The following controls for RNS60 were also used in this study:

- NS, normal saline from the same manufacturing batch. This saline contacted the same device surfaces as RNS60 and was bottled in the same way.
- RNS10.3, a saline that was processed with TCP flow in the absence of any excess oxygen.
- PNS60, saline with the same oxygen content as RNS60 (55 ± 5 ppm) that was prepared inside of the same device but was not processed with TCP flow.

Careful analysis demonstrated that all four fluids were chemically identical (Khasnavis et al. 2012). Liquid chromatography quadrupole time-of-flight mass spectrometric analysis also showed no difference between RNS60 and other control solutions (Khasnavis et al. 2012). Atomic force microscopy showed that RNS60 displayed different surface nanobubble nucleation characteristics relative to that of control saline solutions (Khasnavis et al. 2012) even when they contained similar levels of dissolved oxygen.

### RNS60/NS Treatment of Mice

Mice were treated with RNS60 or NS (300 μl/mouse/d) via intraperitoneal (i.p.) injection from 3 h after the last injection of MPTP for 7 days as described (Khasnavis et al. 2014).

### Antibodies

Rabbit polyclonal anti-PGC1α, mouse monoclonal anti-Nrf1 and rabbit polyclonal anti-TFAM antibodies were obtained from Abcam (Cambridge, MA). Anti-tyrosine hydroxylase (TH), anti-p300 and anti-NeuN antibodies were obtained Millipore (Billerica, MA). Rabbit monoclonal antibodies against phospho-CREB, total CREB and CREB-binding protein (CBP) were purchased from Cell Signaling (Beverly, MA). Cy2- and Cy5-conjugated antibodies were obtained from Jackson Immuno Research Laboratories (West Grove, PA).

### Assay of PI3K Activation

Upon activation, PI3K subunits translocate to the plasma membrane. Therefore, after different time of treatment with RNS60 and NS, subunits of PI3K were monitored in plasma membrane. Briefly, cells were dissociated in 100 mM sodium bicarbonate buffer (pH 11.5) and spun in an ultracentrifuge at 40,000 rpm for 1 h at 4°C. The resultant supernatant was aspirated and the pellet was immersed in double-distilled water and SDS and stored at −80°C overnight. The following day, the pellet was resuspended by repeated grinding and boiling, and processed for Western blot using antibodies against p110α, p110β and p110γ (Khasnavis et al. 2014).

### Western Blot Analysis

Western blotting was conducted as described earlier (Corbett et al.; Saha et al. 2006) with modifications. Briefly, cells were scraped in 1X RIPA buffer, protein was measured using Bradford reagent and sodium dodecyl sulfate (SDS) buffer was added and electrophoresed on NuPAGE® Novex® 4–12% Bis-Tris gels (Life Technologies, Grand Island, NY) and proteins transferred onto a nitrocellulose membrane (Bio-Rad) using the Thermo-Pierce Fast Semi-Dry Blotter. The membrane was then washed for 15 min in TBS plus Tween 20 (TBST) and blocked for 1 h in TBST containing BSA. Next, membranes were incubated overnight at 4°C under shaking conditions with primary antibodies. The next day, membranes were washed in TBST for 1 h, incubated in secondary antibodies against for 1 h at room temperature, washed for one more hour and visualized under the Odyssey® Infrared Imaging System (Li-COR, Lincoln, NE).

### Semi-Quantitative RT-PCR Analysis

Total RNA was isolated from ventral midbrain using Ultraspec-II RNA reagent (Biotecx Laboratories, Inc., Houston, TX) following the manufacturer’s protocol. To remove any contaminating genomic DNA, total RNA was digested with DNase. RT-PCR was carried out as described earlier (Jana et al. 2007; Brahmachari et al. 2009; Ghosh et al. 2009) using a RT-PCR kit (Clontech, Mountain View, CA) and the following primers:

- NRF1: Sense: 5′-CTTCATGGAGGAGCACGGAG-3′

Antisense: 5′-ACTGTGCTCAGTGTACGTGG-3′

- TFAM: Sense: 5′-CAGTAGCCTTGTGGGCTTTC-3′

Antisense: 5′-CTGCCATGTGTTCTCCTGGG-3′

- TOMM20: Sense: 5′-GTGCCATCTTGACGGGAGAT-3′

Antisense: 5′-GCACTGATGCAAGTGAGCTG-3′

- MCU: Sense: 5′-GCCACCAAAGAGAGACCTCC-3′

Antisense: 5′-GCTCAATGCACAGTGTGGTG-3′

- PGC1α: Sense: 5′-TCTCAGTAAGGGGCTGGTTG-3′

Antisense: 5′-TTCCGATTGGTCGCTACACC-3′

- TFEB: Sense: 5′-AAC AAA GGC ACC ATC CTC AA -3′

Antisense: 5′-CAG CTC GGC CAT ATT CAC AC-3′

- LAMP2: Sense: 5′-GGT GCT GGT CTT TCA GGC TTG ATT-3′

Antisense: 5′- ACC ACC CAA TCT AAG AGC AGG ACT-3′

- GAPDH: Sense: 5′-GGTGAAGGTCGGTGTGAACG-3′

Antisense: 5′-TTGGCTCCACCCTTCAAGTG-3′.

### Real-Time PCR Analysis

The mRNA quantification was performed using the ABI-Prism7700 sequence detection system (Applied Biosystems, Foster City, CA) using SYBR Select master mix (Applied Biosystems). The mRNA expression of the targeted genes was normalized to the level of *Gapdh* mRNA and data was processed by the ABI Sequence Detection System 1.6 software as described earlier (Jana et al. 2007; Brahmachari et al. 2009; Ghosh et al. 2009; Khasnavis and Pahan 2012).

### Quantification of Mitochondrial DNA (mtDNA)

Total DNA was isolated from MN9D cells using QIAamp DNA mini kit (Qiagen). The mtDNA content relative to nuclear DNA was assessed by real-time PCR using the ABI7500 (Applied Biosystems).

Primers of the mtDNA encoded *Cox1* gene:

- Forward: 5′-TGCTAGCCGCAGCATTAC-3′
- Reverse: 5′-GGGTGCCCAAAGAATCAGAAC-3′
- Primers of the single copy nuclear gene *Ndufv1*:
- Forward: 5′-CTTCCCCACTGGCCTCAAG-3′
- Reverse: 5′-CCAAAACCCAGTGATCCAGC-3′.
- Relative mtDNA was determined using ΔC_T_ method.

### Monitoring Mitochondrial Membrane Potential

ᅟ

### By Microplate Reader

Briefly, cells were plated in 96-well microplates and treated with RNS60 and NS for 18 h under serum-free condition. Cells were incubated with 500 nM TMRE for 30 min at 37°C. Media was replaced with 100 uL PBS/0.2% BSA. Plate was read on a PerkinElmer fluorescence plate reader with Ex/Em 549/575 nm range.

### By FACS Analysis

Briefly, 10^5^ cells were plated and treated with RNS60 and NS for 18 h under serum-free condition. Cells were incubated with 100 nM TMRE for 30 min at 37°C. Media was replaced with PBS/0.2% BSA for flow cytometry analysis. TMRE signal was detected in PE-FL2 channel in a BD FACSCanto II Flow Cytometer.

### Chromatin Immunoprecipitation Assay

ChIP assays were performed as described earlier by us with a few modifications (Roy et al. 2013; Roy et al. 2015). Briefly, after treatment, cells were fixed with formaldehyde (1.42% final volume) and quenched with 125 mM glycine. The cells were then pelleted and lysed in IP buffer containing 150 mM NaCl, 50 mM Tris-HCl (pH 7.5), 5 mM EDTA, NP-40 (0.5% vol/vol), Triton X-100 (1.0% vol/vol). For 500 ml, add 4.383 g NaCl, 25 ml of 100 mM EDTA (pH 8.0), 25 ml of 1 M Tris-HCl (pH 7.5), 25 ml of 10% (*v/v*) NP-40 and 50 ml of 10% (*v/v*) Triton X-100 containing the following inhibitors; 10 μg/ml leupeptin, 0.5 mM phenylmethlysulfonyl fluoride (PMSF), 30 mM p-nitrophenyl phosphate, 10 mM NaF, 0.1 mM Na_3_VO_4_, 0.1 mM Na_2_MoO_4_ and 10 mM β-glycerophosphate. After one wash with 1.0 ml IP buffer, the pellet was resuspended in 1 ml IP buffer (containing all inhibitors) and sonicated and sheared chromatin was split into two fractions (one to be used as Input). The remaining fraction was incubated overnight under rotation at 4°C with 3-4 μg of anti-CREB, anti-CBP or anti-p300 antibodies followed by incubation with Protein G-Agarose (Santa Cruz) for 2 h at 4°C under rotation. Normal IgG was also run as control. Beads were then washed five times with cold IP buffer and a total of 100 μl of 10% Chelex (10 g/100 ml H_2_O) was added directly to the washed protein G beads and vortexed. After 10 min boiling, the Chelex/protein G bead suspension was allowed to cool to room temperature. Proteinase K (100 μg/ml) was then added and beads were incubated for 30 min at 55°C while shaking, followed by another round of boiling for 10 min. The suspension was centrifuged and supernatant collected. The Chelex/protein G beads fraction was vortexed with another 100 μl water, centrifuged again, and the first and the second supernatants were combined. Eluate was used directly as a template in PCR. The following primers were used to amplify fragments flanking CRE in the mouse *Pgc1α* promoter:

- Sense: 5′-GCGTTACTTCACTGAGGCAG-3′
- Antisense: 5′-CAGCCTCCCTTCTCCTGTGC-3′

The PCRs were repeated by using varying cycle numbers and different amounts of templates to ensure that results were in the linear range of PCR. Quantitative real-time PCR was also performed using the same primers and SYBR select MasterMix. Data were normalized to input and nonspecific IgG, and fold increase versus control was calculated.

### Immunostaining of Cells

Immunocytochemistry was performed as described earlier (Khasnavis and Pahan). Briefly, after treatment, cells were fixed with chilled Methanol (Fisher Scientific, Waltham, MA) overnight, followed by two brief rinses with filtered PBS. Samples were blocked with 2% BSA (Fisher Scientific) in PBS containing Tween 20 (Sigma) and Triton X-100 (Sigma) for 30 min and incubated at room temperature under shaking conditions for 2 h in PBS containing the following anti-mouse primary antibodies: TFAM (1:500), PGC1α (1:300), Nrf1 (1:300), and NeuN (1:500). After four 15 min washes in filtered PBS, the slides were further incubated with Cy2 or Cy5-labeled secondary antibodies (all 1:200; Jackson ImmunoResearch, West Grove, PA) for 1 h under similar shaking conditions. Following four 15 min washes with filtered PBS, cells were incubated for 4–5 min with 4′, 6-diamidino-2-phenylindole (DAPI, 1:10,000; Sigma). The samples were run in an EtOH and Xylene (Fisher) gradient, mounted, and observed under Olympus BX41 fluorescence microscope.

### Immunostaining of Tissue Sections

After treatment, mice were sacrificed and their brains fixed, embedded, and processed. Sections were made from ventral midbrain regions and for immunofluorescence staining on fresh frozen sections, anti-mouse TFAM (1:300), anti-mouse Nrf1 (1:200), anti-mouse PGC1α (1:200), and anti-mouse NeuN (1:500) were used (Ghosh et al. 2015). The samples were mounted and observed under Olympus BX41 fluorescence microscope (Dasgupta et al. 2004).

### Electron Microscopy and Counting of Mitochondria and Lysosomes

After treatment, cells were fixed with paraformaldehyde (2%) and glutaraldehyde (2.5%) mixture. After primary fixation, samples were prepared in the Electron Microscopy core facility of the University of Illinois at Chicago Research Resources Center. To stabilize cell components, samples were treated with 1% osmium tetroxide in phosphate buffer. Next, the samples were dehydrated through an increasing concentration of ethanol, passed through propylene oxide and then infiltrated and embedded in a liquid resin. Resin block is then sectioned by ultramicrotomy with 50–70 nm thickness and collected on metal mesh ‘grids’ followed by staining these grids with electron dense stains before observation in the TEM (JEOL JEM-1220).

Counting analysis was performed using Olympus Microsuite V software with the help of a touch counting module. Before counting cells, the entire image area was calibrated with the help of a rectangular box available in the touch counting panel. Once the area of the image was measured, the touch counting program was applied to count the number of mitochondria or lysosomes using a simple mouse click method. Next, the total number of signals in a given area was normalized by the total area of the image and presented as number of cells per square millimeter unit. Counting was done for 20 fields in each treatment group.

### MitoTracker Staining

Cells cultured to 70–80% confluence were subjected to different stimuli under serum-free condition followed by incubation with 75 nM MitoTracker Red (Thermo Fisher Scientific, Waltham, MA) for 45mins. Cells were then washed thoroughly with filtered PBS and mounted on glass slides and viewed under BX41 fluorescence microscope. Cells were visualized with a 100× objective and MitoTracker signals were counted as described above using Olympus Microsuite V software.

### Statistics

All values are expressed as means ± SEM. One-way ANOVA was performed while analyzing dose-dependent effect of RNS60 on mRNA expression of *Nrf1*, *Tfam*, *Mcu*, and *Tom20* in neuronal cells. In other cases, Student’s *t*-test was used to compare outcome between two groups (e.g. control vs MPTP, MPTP vs RNS60 etc.).

## Results

### RNS60 Increases the Biogenesis of Mitochondria, but Not Lysosomes, in MN9D Dopaminergic Neuronal Cells

**Fig. 1:**
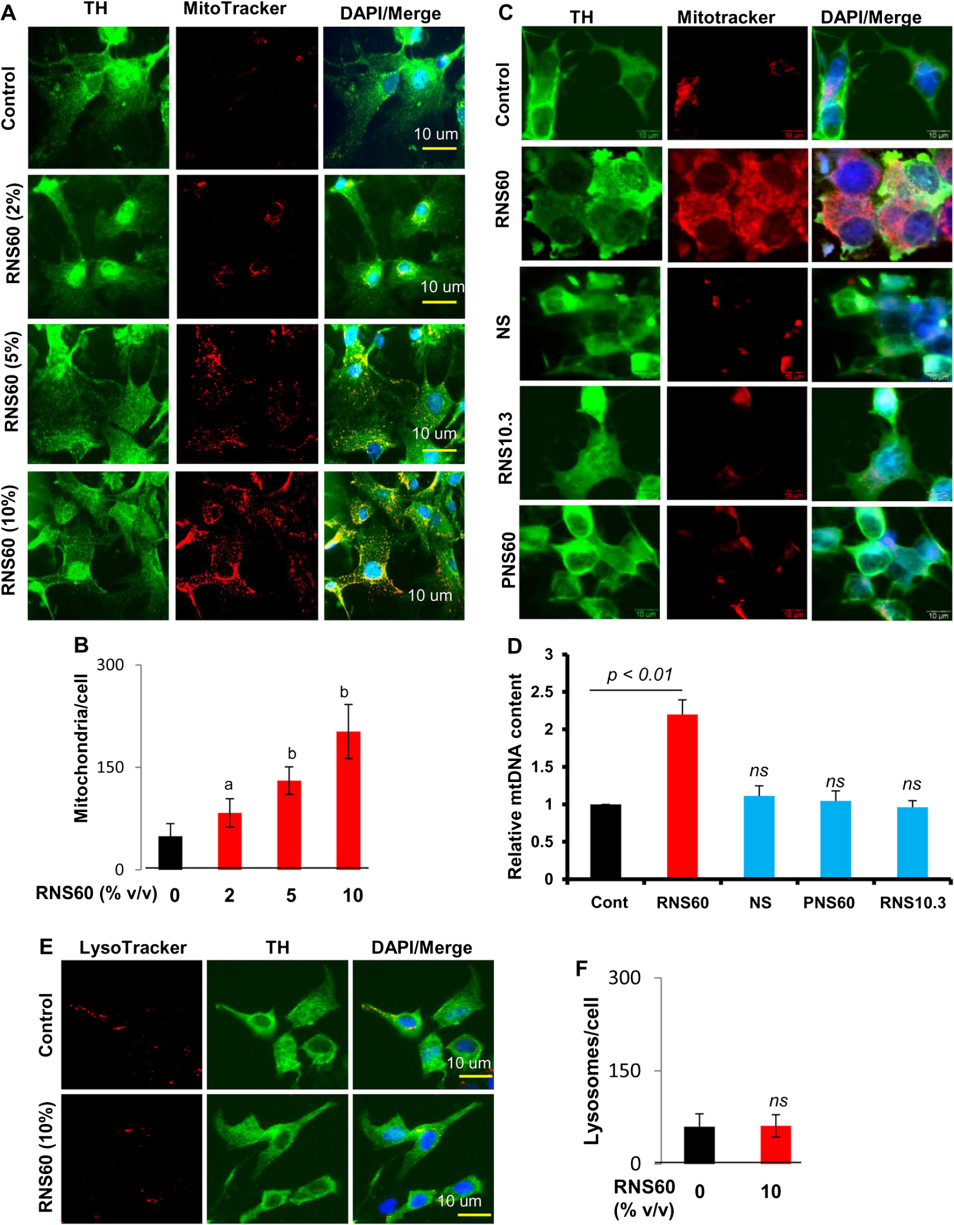
**RNS60 stimulates mitochondrial biogenesis in MN9D mouse neuronal cells**. **a** Cells were treated with different concentrations of RNS60 under serum-free condition for 24 h followed by MitoTracker labeling and immunostaining with anti-TH antibody. **b** Number of mitochondria was counted in 25 different cells and expressed as mitochondria per cell. Data are mean ± SEM of 25 different cells per group. ^*a*^ *p* < 0.05 versus control; ^*b*^ *p* < 0.001 versus control. **c** Cells were treated with 10% *v/v* RNS60, NS, RNS10.3, or PNS60 under serum-free condition for 24 h followed by MitoTracker labeling and immunostaining with anti-TH antibody. **d** Under same treatment condition, mitochondrial DNA content was monitored. **e** Cells were treated with 10% *v/v* RNS60 under serum-free condition for 24 h followed by LysoTracker labeling and immunostaining with anti-TH antibody. **f** Number of lysosomes was counted in 25 different cells and expressed as lysosomes per cell. Data are mean ± SEM of 25 different cells per group

Increasing mitochondrial biogenesis in neurons may have implications in neurodegenerative disorders. Therefore, we first investigated whether RNS60 containing charge-stabilized nanostructures (Khasnavis et al. 2012; Khasnavis et al. 2014) could upregulate mitochondrial biogenesis in MN9D neuronal cells. Cells treated with RNS60 for 24 h under serum-free condition were double-labeled with MitoTracker and tyrosine hydroxylase (TH). Although RNS60 did not modulate the level of TH, we found dose-dependent increase in mitochondrial content per cell after RNS60 treatment (Fig. 1a–b). On the other hand, unprocessed NS from the same manufacturing batch had no such stimulatory effect on mitochondrial biogenesis (Fig. 1c). Furthermore, RNS10.3, saline that was processed with TCP flow in the absence of any excess oxygen, and PNS60, saline with same oxygen content as RNS60 (55 ± 5 ppm) that was prepared inside of the same device but was not processed with TCP flow (Khasnavis et al. 2012), also remained unable to stimulate mitochondrial content in neuronal cells (Fig. 1c). To confirm this finding further, we also monitored mitochondrial DNA. Similar to MitoTracker labeling, RNS60, but not NS, RNS10.3 and PNS60, increased mitochondrial DNA in MN9D neuronal cells (Fig. 1d). These results suggest that stimulation of mitochondrial biogenesis in neuronal cells is specific to RNS60.

**Fig. 2:**
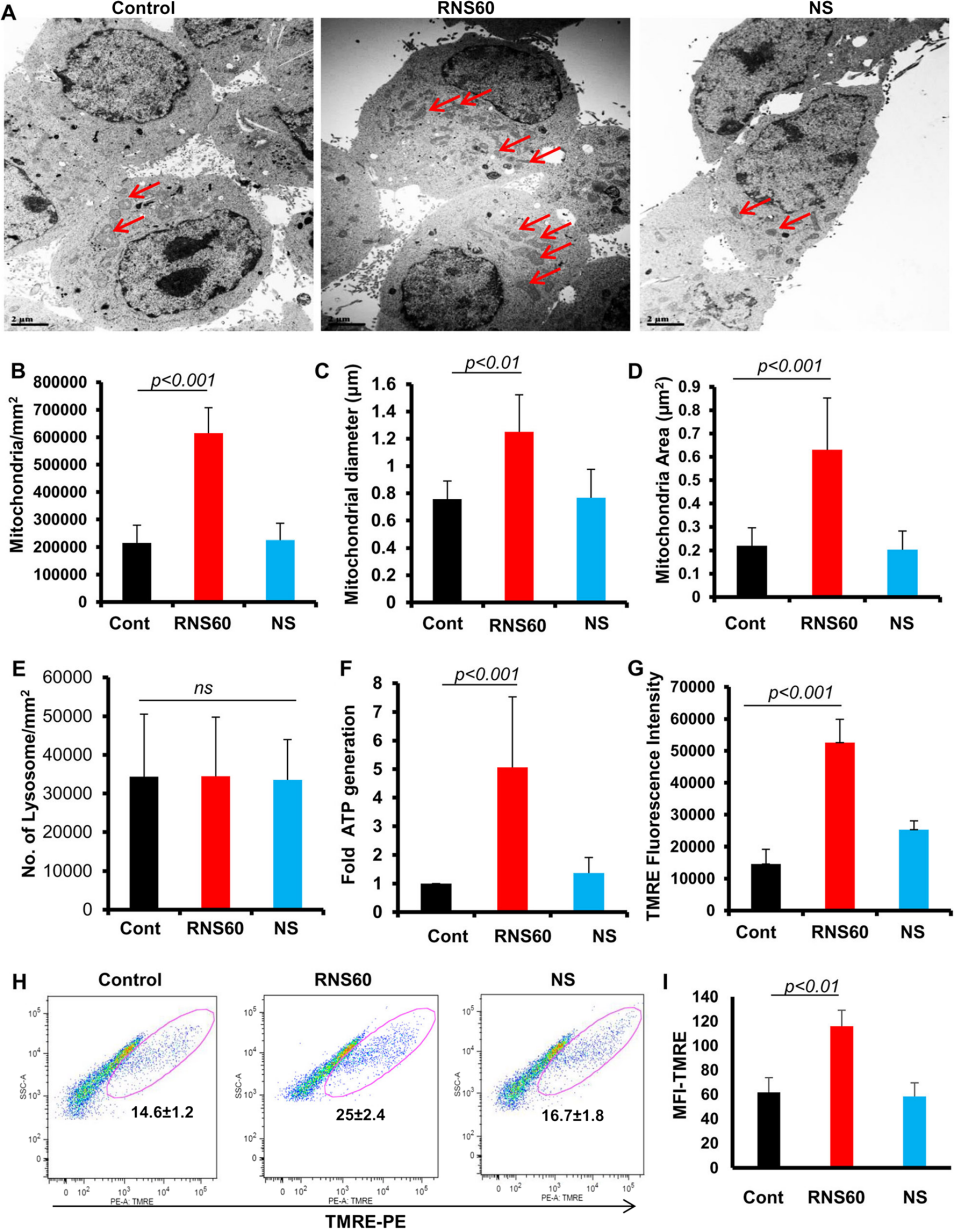
**Electron microscopic detection of mitochondria and determination of mitochondrial membrane potential in RNS60-treated MN9D mouse neuronal cells**. **a** Cells were treated with RNS60 (10% *v/v*) and NS (10% *v/v*) under serum-free condition for 24 h and processed for electron microscopy as described under “Materials and Methods”. Number of mitochondria (**b**) was counted using Olympus Microsuite V software with the help of a touch counting module. Results are mean ± SEM of 20 fields. Mean mitochondrial diameter _max_ (**c**) and area (**d**) was determined by measuring at least 80 individual mitochondria from images of multiple cells using Olympus Microsuite V software. Number of lysosomes (**e**) was counted using Olympus Microsuite V software. Results are mean ± SEM of 20 fields. NS, not significant. **f** ATP production was measured using the Luminescent ATP detection kit (Abcam). Data are mean ± S.D. of three independent experiments. **g** After 30 min of incubation with 500 nM TMRE, mitochondrial membrane potential was monitored in a fluorescent plate reader. Results are mean ± S.D. of three independent experiments. **h** TMRE signal was detected by FACS analysis. **i** Mean fluorescent intensity (MFI) of TMRE was calculated by using the CellQuest software. Results are mean ± S.D. of three independent experiments

Next, we monitored the effect of RNS60 on lysosomal biogenesis to test if RNS60’s effects are organelle-specific. Under the same treatment condition as described above, cells were double-labeled with LysoTracker and TH. In contrast to the stimulation of MitoTracker labeling by RNS60, we did not see any increase in LysoTracker staining in RNS60-treated neuronal cells (Fig. 1e–f). To confirm these finding further, we performed electron microscopy (EM) of MN9D neuronal cells after RNS60 and NS treatment. Similar to MitoTracker labeling, RNS60, but not NS, upregulated the number of mitochondria (Fig. 2a–b) and increased mitochondrial diameter (Fig. 2c) and area (Fig. 2d) in MN9D neuronal cells. On the other hand, either RNS60 or NS had no effect on lysosomal biogenesis (Fig. 2a and e). Consistent to increase in mitochondrial number, shape and size, RNS60, but not NS, also increased ATP production (Fig. 2f) and stimulated mitochondrial membrane potential (Fig. 2g–i) in MN9D neuronal cells.

### RNS60 Increases the Expression of Genes Associated with Mitochondrial Biogenesis in MN9D Dopaminergic Neuronal Cells

**Fig. 3:**
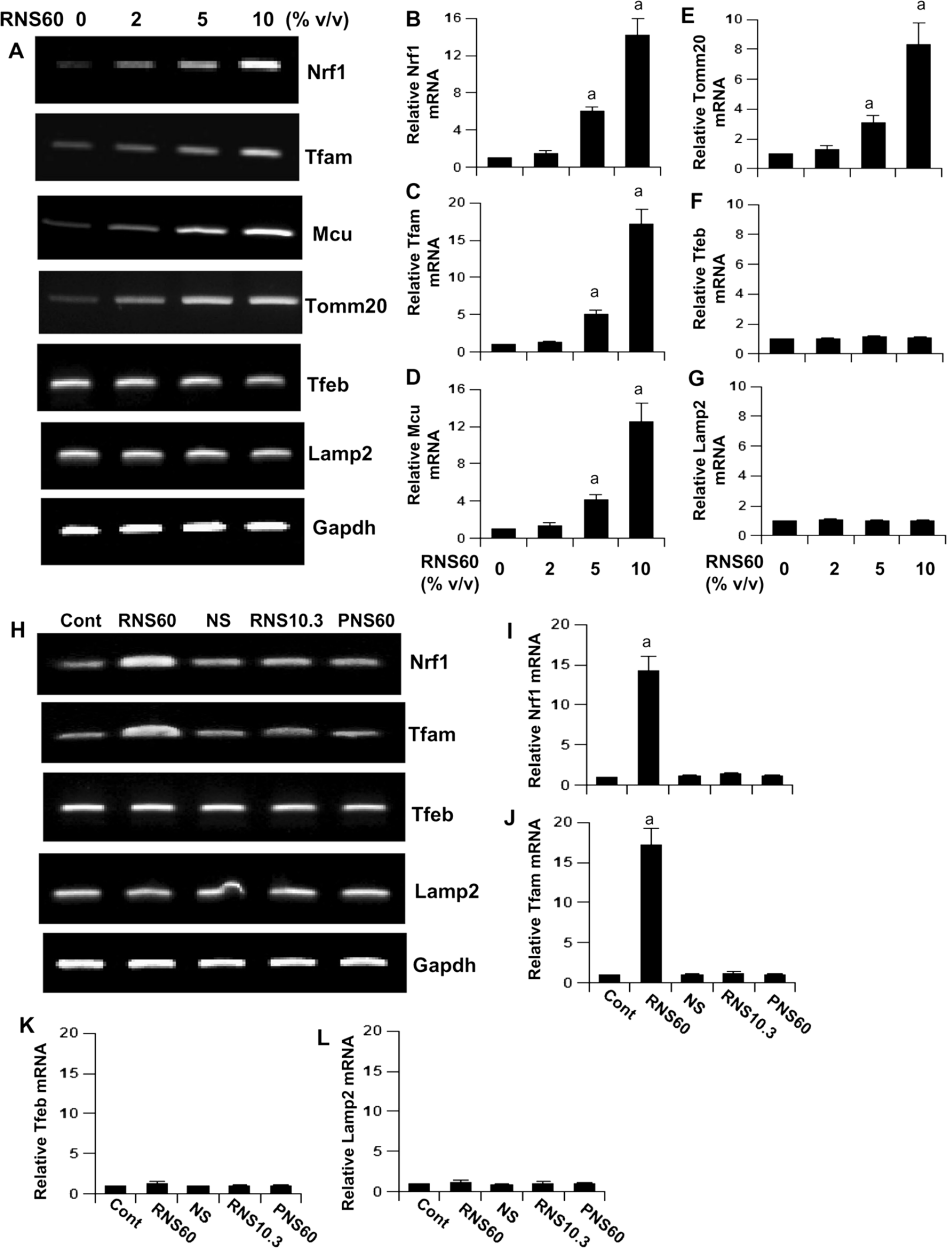
**RNS60 upregulates the expression of genes associated with mitochondrial biogenesis in MN9D mouse neuronal cells**. Cells were treated with different concentrations of RNS60 for 5 h followed by monitoring the mRNA expression of Nrf1, Tfam, Mcu, Tomm20, Tfeb, and Lamp2 by RT-PCR (**a**) and real-time PCR (**b**, Nrf1; **c**, Tfam; **d**, Mcu; **e**, Tomm20; **f**, Tfeb; **g**, Lamp2). Results are mean ± S.D. of three independent experiments. ^*a*^ *p* < 0.001 versus control. Cells were treated with 10% *v/v* RNS60, NS, RNS10.3, and PNS60 for 5 h followed by monitoring the mRNA expression of Nrf1, Tfam, Tfeb, and Lamp2 by RT-PCR (**h**) and real-time PCR (**i**, Nrf1; **j**, Pgc1a; **k**, Tfam; **l**, Tfeb). Results are mean ± S.D. of three independent experiments. ^*a*^ *p* < 0.001 versus control

Although mitochondria has its own genome, the majority of mitochondrial proteins are encoded in the nucleus. For example, nuclear respiratory factor 1 is linked to the transcriptional control of many mitochondrial genes. Mitochondrial transcription factor A (TFAM), the transcription factor controlling the transcription of mitochondrial genes (Kukat and Larsson 2013), is also transcribed in the nucleus. Therefore, we examined if RNS60 could modulate the expression of genes responsible for mitochondrial biogenesis. It is clearly evident from Fig. 3a that RNS60 dose-dependently increased the mRNA expression of *Nrf1* and *Tfam* as well as *Tomm20* and *Mcu*, genes that encode mitochondrial membrane proteins translocase of outer mitochondrial membrane 20 (TOMM20) and mitochondrial calcium uniporter (MCU), respectively (Schleiff and Turnbull 1998; Petrungaro et al. 2015). These results are also supported by real-time PCR analysis (Fig. 3b–e). On the other hand, consistent to LysoTracker results, RNS60 was unable to stimulate the expression of *Tfeb* and *Lamp2*, genes that are responsible for lysosomal biogenesis, at any of the doses tested (Fig. 3f–g). To understand the specificity, cells were also treated with NS, RNS10.3 and PNS60. Similar to MitoTracker results, NS, RNS10.3 and PNS60 remained unable to stimulate the expression of *Nrf1* and *Tfam* in MN9D neuronal cells (Fig. 3h–j), indicating that the induction of *Nrf1* and *Tfam* is specific for RNS60. As expected, NS, RNS10.3 and PNS60 also did not modulate the expression of *Tfeb* and *Lamp2* (Fig. 3k–l).

### RNS60 Increases Mitochondrial Biogenesis in Primary Mouse Dopaminergic Neurons

**Fig. 4:**
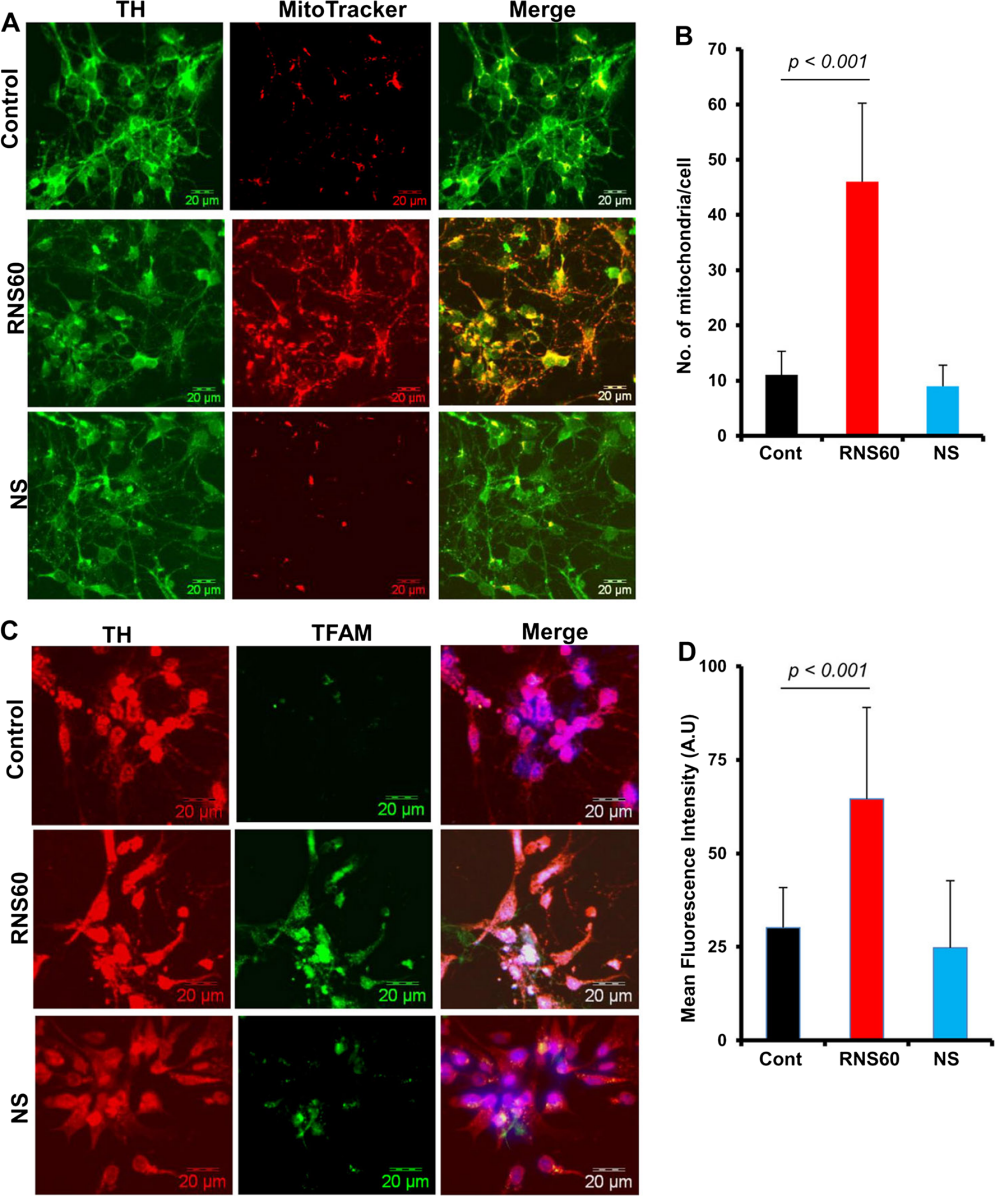
**RNS60 treatment upregulates mitochondrial biogenesis in mouse primary dopaminergic neurons**. Neurons isolated from ventral mesencephalon of E12.5 to E14 d old fetus were treated with 10% *v/v* RNS60 or NS for 24 h followed by MitoTracker staining and immunostaining with anti-TH antibody (**a**). Number of mitochondria was counted in 25 different cells (**b**) and expressed as mitochondria per cell. Data are mean ± SEM of 25 different cells per group. **c**) Under similar treatment condition, cells were double-labeled for TH and TFAM. **d**) Mean Fluorescence Intensity of TFAM was measured in 10 different images using the measurement module of microsuite V Olympus software. Data are mean ± SEM of 10 different images per group

Results seen in a transformed cell line are often not reflective of events in primary cells. Therefore, next, we investigated whether RNS60 was able to induce mitochondrial biogenesis in primary dopaminergic neurons. Cells treated with RNS60 and NS for 24 h under serum-free condition were double-labeled for MitoTracker and TH. Similar to MN9D neuronal cells, RNS60, but not NS, increased MitoTracker labeling in dopaminergic neurons (Fig. 4a–b). Again, we did not see any change in TH expression by RNS60 treatment (Fig. 4a). To confirm these findings further, cells were double-labeled for TFAM and TH after RNS60 treatment. In this case as well, RNS60, but not NS, increased the level of TFAM in dopaminergic neurons (Fig. 4c–d). These results suggest that RNS60 is capable of stimulating mitochondrial biogenesis in primary dopaminergic neurons.

### RNS60 Treatment Protects and/or Stimulates Mitochondrial Biogenesis in the Nigra of MPTP-Intoxicated Mice

**Fig. 5:**
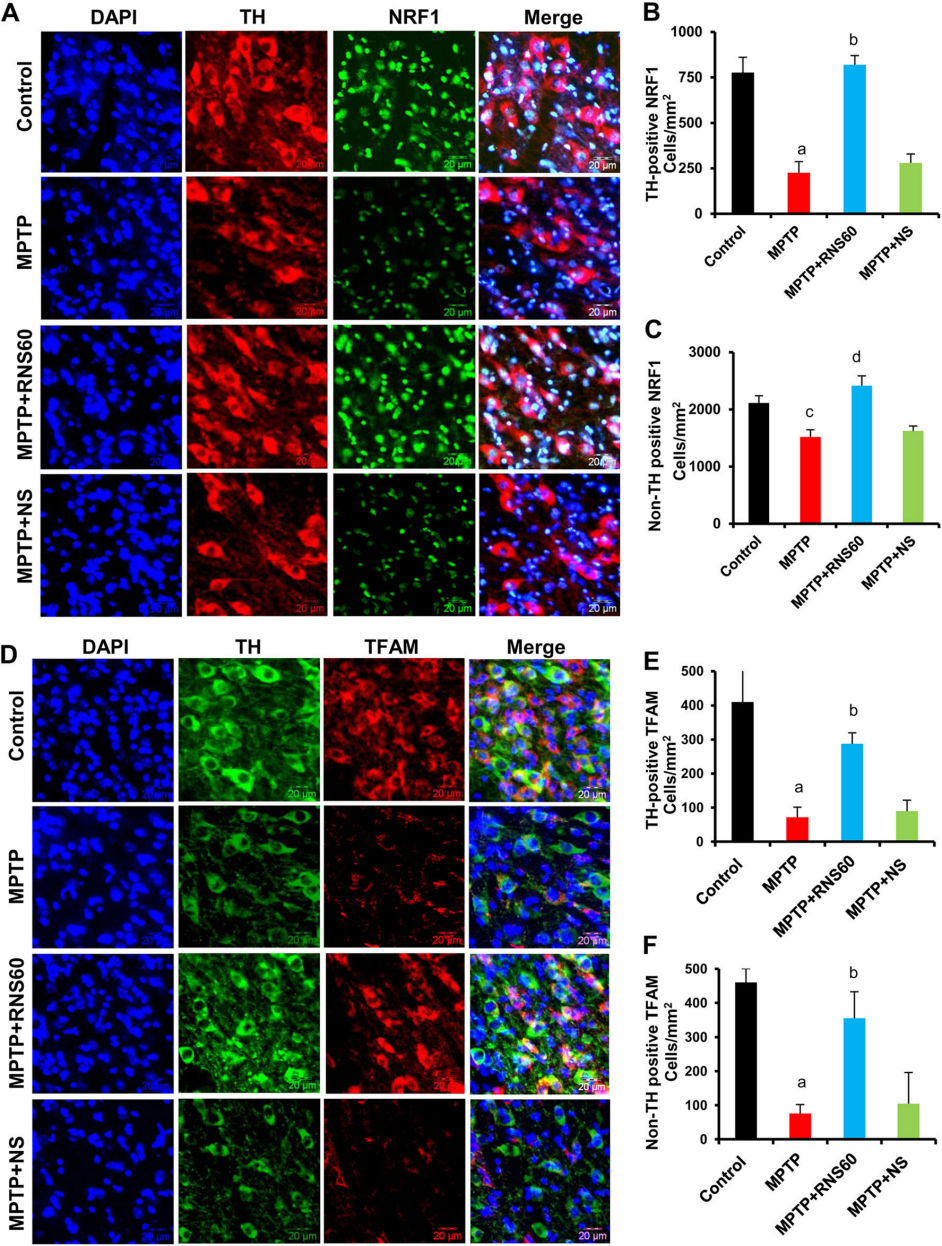
**RNS60 treatment upregulates mitochondrial biogenesis in vivo in the nigra of MPTP-intoxicated mice**. Mice receiving either RNS60 or NS (300 μl/mouse/d) via i.p. injection from 3 h after the last injection of MPTP were sacrificed 7 days after the last injection of MPTP followed by double-labeling of nigral sections with antibodies against either TH & NRF1 (**a**) or TH & TFAM (**d**). Cells positive for NRF1 and TFAM (**b**, TH positive NRF1, **c**; Non TH positive NRF1; **e**, TH positive TFAM; **f**, Non TH positive TFAM) were counted in two nigral sections (2 images per slide) from each of five different mice. ^*a*^ *p* < 0.001 versus control; ^*b*^ *p* < 0.001 versus MPTP

Due to the lack of a proper detection tool to detect nanobubble-based structures, at present, we do not have any direct way to measure RNS60 within the CNS. However, within 3 h of intraperitoneal administration of RNS60, but not NS, we have observed the activation of class IA PI3K and the upregulation of IκBα, signature events of RNS60 (Khasnavis et al. 2012), in vivo in the nigra (Khasnavis et al. 2014). Accordingly, intraperitoneal administration of RNS60, but not NS, also protects dopaminergic neurons in MPTP mouse model of PD (Khasnavis et al. 2014). Therefore, it is likely that RNS60 enters the brain and we examined if RNS60 could modulate mitochondrial biogenesis in the nigra of MPTP-intoxicated mice. Mice received either RNS60 or NS (300 μl/mouse/d) via intraperitoneal (i.p.) injection from 3 h after the last injection of MPTP and after 7 days of MPTP insult, mitochondrial biogenesis was monitored in nigral sections by double-labeling of TH & NRF1 (Fig. 5a–b) and TH & TFAM (Fig. 5c–d). In control nigra, both NRF1 and TFAM were present in TH-positive neurons as well as TH-negative cells (Fig. 5). MPTP intoxication led to a decrease in mitochondrial content in the nigra as compared to control, which is evident from decrease in NRF1 (Fig. 5a–b) and TFAM (Fig. 5c–d) in the nigra. However, RNS60, but not NS, treatment led to the restoration of both NRF1 (Fig. 5a–b) and TFAM (Fig. 5c–d) in the nigra of MPTP-insulted mice.

### Upregulation of PGC1α in Dopaminergic Neurons by RNS60

**Fig. 6:**
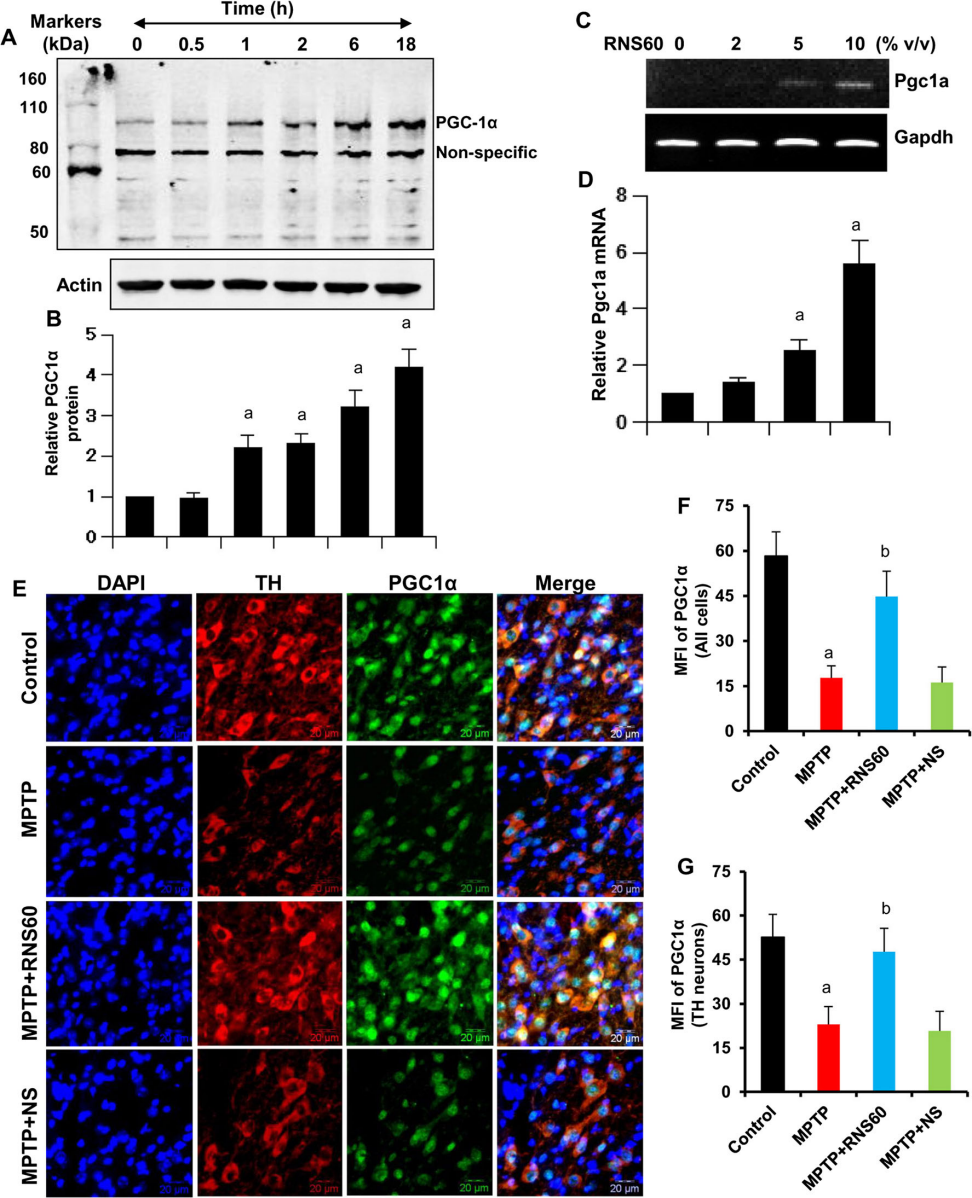
**Upregulation of PGC1α by RNS60**. MN9D cells were treated with 10% *v/v* RNS60 under serum-free condition for different time intervals followed by monitoring the level of PGC1α by Western blot (**a**). Actin was run as loading control. Bands were scanned and values (PGC1α/Actin) presented as relative to control (**b**). Results are mean ± SD of three different experiments. ^*a*^ *p* < 0.05 versus control; ^*b*^ *p* < 0.001 versus control. MN9D cells were treated with different concentrations of RNS60 for 2 h followed by monitoring the mRNA expression of *Pgc1a* by RT-PCR (**c**) and real-time PCR (**d**). Mice receiving either RNS60 or NS (300 μl/mouse/d) via i.p. injection from 3 h after the last injection of MPTP were sacrificed 7 days after the last injection of MPTP followed by double-labeling of nigral sections with antibodies against TH & PGC1α (**e**). Mean fluorescence intensity (MFI) of PGC1α in all cells (**f**) and TH-positive neurons (**g**) were calculated in two nigral sections (10 cells per section) from each of five different mice using the “measurement module” of Olympus Microsuite V software. ^*a*^ *p* < 0.001 versus control; ^*b*^ *p* < 0.001 versus MPTP

Since PPARγ coactivator 1α (PGC1α) plays an important role in the biogenesis of mitochondria (Wareski et al. 2009), in order to understand the mechanisms behind RNS60-mediated stimulation of mitochondrial biogenesis, we examined the effect of RNS60 on PGC1α. RNS60, but not NS, treatment rapidly increased the expression of PGC1α protein in MN9D neuronal cells, which was evident as early as 2 h after RNS60 stimulation (Fig. 6a–b). The increase in PGC1α mRNA by RNS60 (Fig. 6c–d) suggests that the upregulation occurs at the transcriptional level. Next we examined the effect of RNS60 on the level of PGC1α in vivo in the nigra of MPTP-insulted mice. Although MPTP intoxication led to a decrease in PGC1α in the nigra compared to control, RNS60 treatment resulted in the restoration and/or upregulation of nigral PGC1α (Fig. 6e–f). This effect was specific as NS treatment had no such effect (Fig. 6e–f).

### RNS60 Increases Mitochondrial Biogenesis in MN9D Dopaminergic Neuronal Cells via PGC1α

**Fig. 7:**
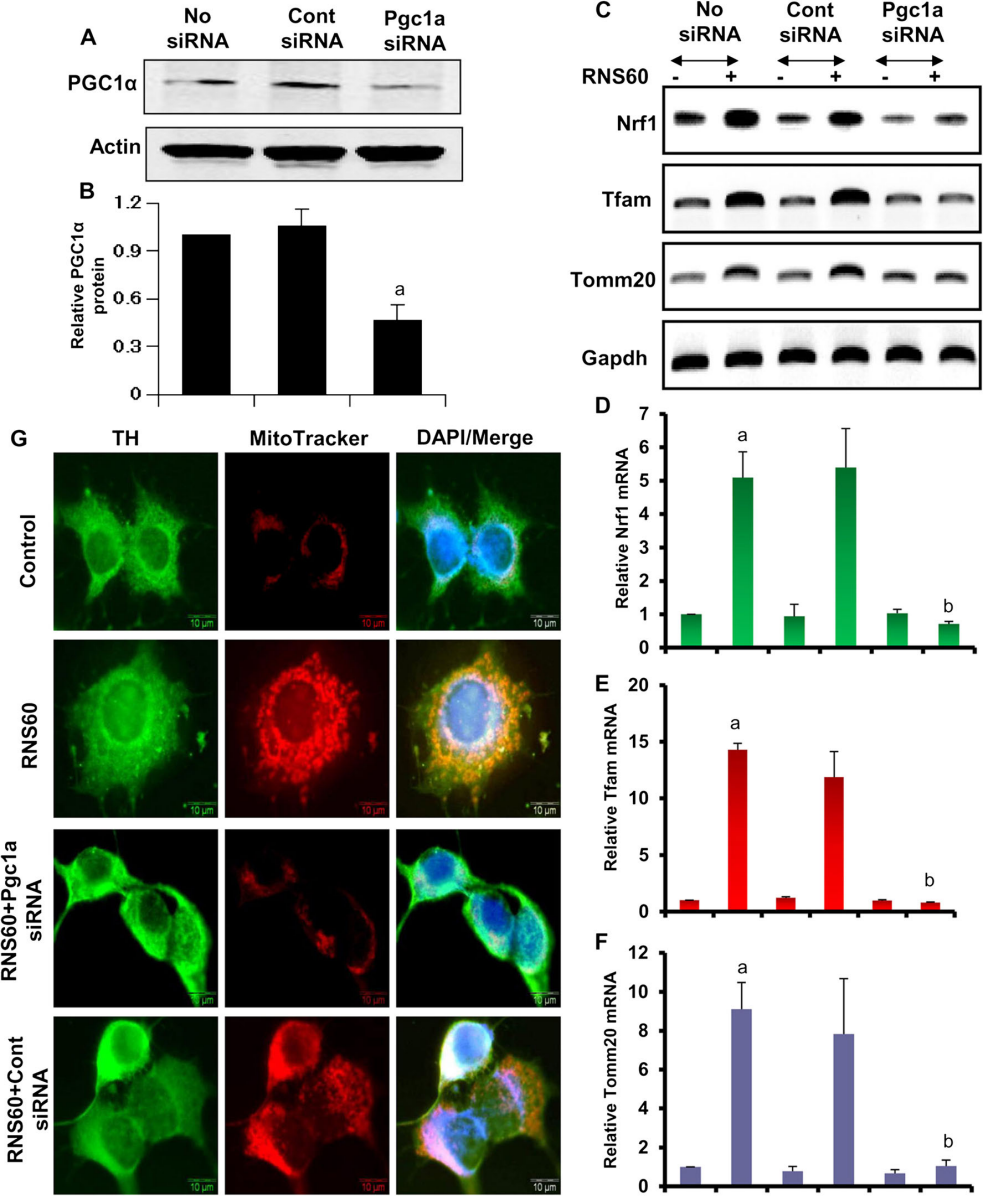
**SiRNA knockdown of PGC1α abrogates RNS60-mediated mitochondrial biogenesis in MN9D mouse neuronal cells**. Cells were transfected with control or PGC1α siRNAs. After 48 h of transfection, cells were treated with 10% *v/v* RNS60 for 4 h followed by monitoring the level of PGC1α by Western blot (**a**). Actin was run as loading control. Bands were scanned and values (PGC1α/Actin) presented as relative to control (**b**). Results are mean ± SD of three different experiments. ^*a*^ *p* < 0.001 versus control; ^*b*^ *p* < 0.001 versus control siRNA-RNS60. After 48 h of transfection, cells were treated with 10% *v/v* RNS60 for 5 h followed by monitoring the mRNA expression of Nrf1, Tfam and Tomm20 by RT-PCR (**c**) and real-time PCR (**d**, Nrf1; **e**, Tfam; **f**, Tomm20). Results are mean ± SD of three different experiments. ^*a*^ *p* < 0.001 versus control; ^*b*^ *p* < 0.001 versus RNS60. **g**) After 48 h of transfection, cells were treated with 10% *v/v* RNS60 for 24 h followed by double-labeling for MitoTracker and TH. Results represent three independent experiments

Since RNS60 induced the level of PGC1α, we examined if induction of PGC1α was essential for the upregulation of mitochondrial biogenesis by RNS60. We employed PGC1α siRNA to knockdown PGC1α in neurons. As expected, PGC1α siRNA, but not control siRNA, suppressed RNS60-mediated increase in PGC1α protein in MN9D cells (Fig. 7a–b). Moreover, siRNA knockdown of PGC1α inhibited RNS60-mediated increase in *Tfam*, *Nrf1*, *Mcu*, and *Tomm20* mRNAs in MN9D neuronal cells (Fig. 7c). These results were confirmed by real-time PCR (Fig. 7d–g). Finally, we also monitored mitochondrial biogenesis. As expected, RNS60 increased MitoTracker labeling in MN9D neuronal cells (Fig. 7g). However, PGC1α siRNA, but not control siRNA, markedly inhibited RNS60-mediated upregulation in MitoTracker labeling (Fig. 7g). These results demonstrate that RNS60 stimulates mitochondrial biogenesis in neuronal cells via PGC1α.

### RNS60 Stimulates the Transcription of Pgc1a in MN9D Dopaminergic Neuronal Cells via Class IA PI3K-Mediated Activation of CREB

**Fig. 8:**
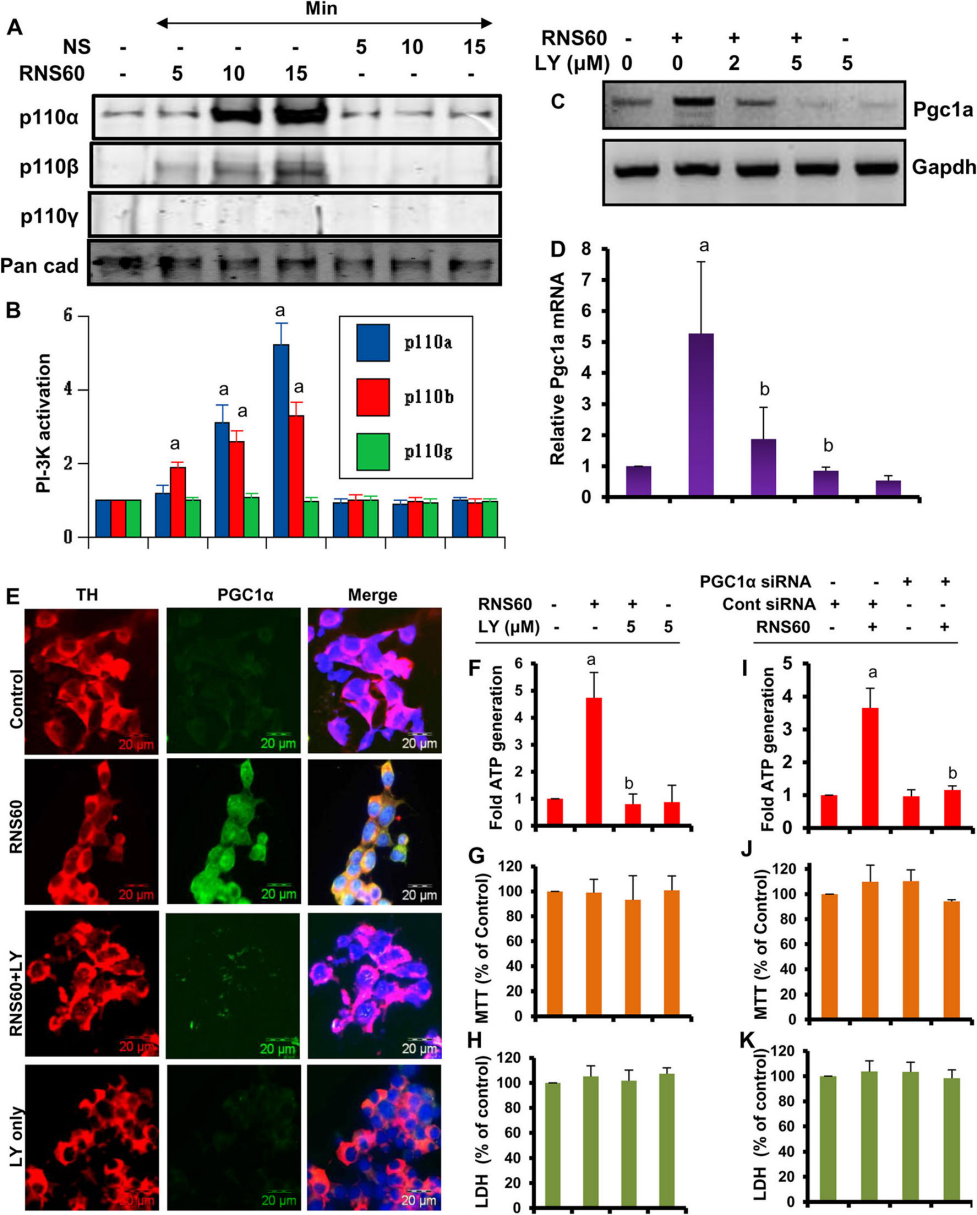
**Role of PI3 kinase in RNS60-mediated upregulation of PGC1α and ATP generation in MN9D mouse neuronal cells**. **a** Cells were treated with 10% *v/v* RNS60 or NS for different minute intervals under serum-free condition followed by monitoring the levels of p110α, p110β and p110γ in cell membranes by Western blot. Pan cadherin was run as a membrane marker. **b** Bands were scanned and values (p110α/pan Cad, p110β/pan Cad and p110γ/pan Cad) are expressed as relative to control. Results are mean ± SD of three different experiments. ^*a*^ *p* < 0.001 versus control. Cells treated with different concentrations of LY294002 (LY) for 30 min were stimulated with 10% *v/v* RNS60 for 2 h min followed by monitoring the mRNA expression of *Pgc1α* by RT-PCR (**c**) and real-time PCR (**d**). Results are mean ± SD of three different experiments. ^*a*^ *p* < 0.001 versus control; ^*b*^ *p* < 0.001 versus RNS60. **e**) Cells treated with 5 μM LY for 30 min were stimulated with 10% *v/v* RNS60 for 6 h followed by double-labeling of TH and PGC1α. Results represent three independent experiments. Cells pretreated with 5 μM LY for 30 min were stimulated with 10% *v/v* RNS60 for 24 h followed by monitoring ATP production (**f**), MTT (**g**) and LDH (**h**). Results are mean ± SD of three different experiments. ^*a*^ *p* < 0.001 versus control; ^*b*^ *p* < 0.001 versus RNS60. Cells were transfected with PGC1α siRNA and control siRNA. After 48 h of transfection, cells were stimulated with 10% *v/v* RNS60 for 24 h followed by monitoring ATP production (**i**), MTT (**j**) and LDH (**k**). Results are mean ± SD of three different experiments. ^*a*^ *p* < 0.001 versus untreated control siRNA; ^*b*^ *p* < 0.001 versus control siRNA-RNS60

Next, we investigated mechanisms by which RNS60 upregulated PGC1α. Earlier we have delineated that RNS60 rapidly activates phosphatidylinositol-3 kinase (PI3K) in microglial cells (Khasnavis et al. 2012). Because PI3K is associated to multiple cellular functions, we examined if PI3K was involved in RNS60-mediated increase in PGC1α in MN9D neuronal cells. At first, we tested the effect of RNS60 on PI3K activation. Class IA PI3K, which is regulated by multiple receptor tyrosine kinases, consists of a heterodimer of a regulatory 85-kDa subunit and a catalytic 110-kDa subunit (p85:p110α/β). On the other hand, class IB PI3K consists of a dimer of a 101-kDa regulatory subunit and a p110γ catalytic subunit (p101/p110γ). Although in resting condition, PI3K subunits are located mainly in cytoplasm, upon activation, these are translocated to the plasma membrane (Franke et al. 1997; Koyasu 2003). Therefore, we monitored the activation of class IA and IB PI3K by the recruitment of p110α, p110β and p110γ to the plasma membrane. Western blotting of membrane fractions for p110 subunits showed that RNS60 specifically induced the recruitment of p110α and p110β, but not p110γ, to the plasma membrane (Fig. 8a). Densitometric analysis of the p110α and p110β at different time points of RNS60 stimulation indicates significant activation of p110α PI3K at 10 and 15 min and p110β PI3K at 5, 10 and 15 min (Fig. 8b). Again these results were specific as NS remained unable to activate p110α and p110β PI3K at any time points tested. Together, these results suggest that RNS60 activates class IA PI3K p110α and p110β, but not class IB PI3K p110γ, in MN9D neuronal cells.

Next, to delineate whether PI3K is involved in RNS60-mediated upregulation of PGC1α, cells were pretreated with different concentrations of LY294002, a morpholine-containing chemical compound that strongly inhibits PI3K, for 30 min followed by stimulation with 10% RNS60 for 2 h and analysis of *Pgc1a* mRNA. As expected, RNS60 treatment increased the mRNA expression of *Pgc1a* (Fig. 8c–d). However, pretreatment with LY294002 abrogated RNS60-mediated increase in *Pgc1a* expression in neuronal cells (Fig. 8c–d). To further confirm, we performed TH:PGC1α double labeling. Although RNS60 did not modulate TH, marked increase in PGC1α protein was seen after RNS60 treatment (Fig. 8e). However, LY294002 strongly suppressed RNS60-mediated upregulation of PGC1α (Fig. 8e). These results suggest that RNS60 upregulates PGC1α in MN9D neuronal cells via PI3K.

Since RNS60 increased the production of ATP (Fig. 2d), we examined if RNS60 required the PI3K-PGC1α pathway for stimulating ATP production in neurons. Suppression of RNS60-induced ATP production by LY294002 suggests the involvement of PI3K in RNS60-mediated ATP generation. Similarly, PGC1α siRNA, but not control siRNA, also reduced the production of ATP in RNS60-stimulated neuronal cells, indicating the involvement of PGC1α in this process. These results were not due to any change in viability as evident from MTT and LDH release assays (Fig. 8g–h and j–k). Although RNS60 protected neurons from Aβ1–42-mediated toxicity (Modi et al. 2014), RNS60 was unable to stimulate the survival of normal neurons (Fig. 8g–h and j–k).

**Fig. 9:**
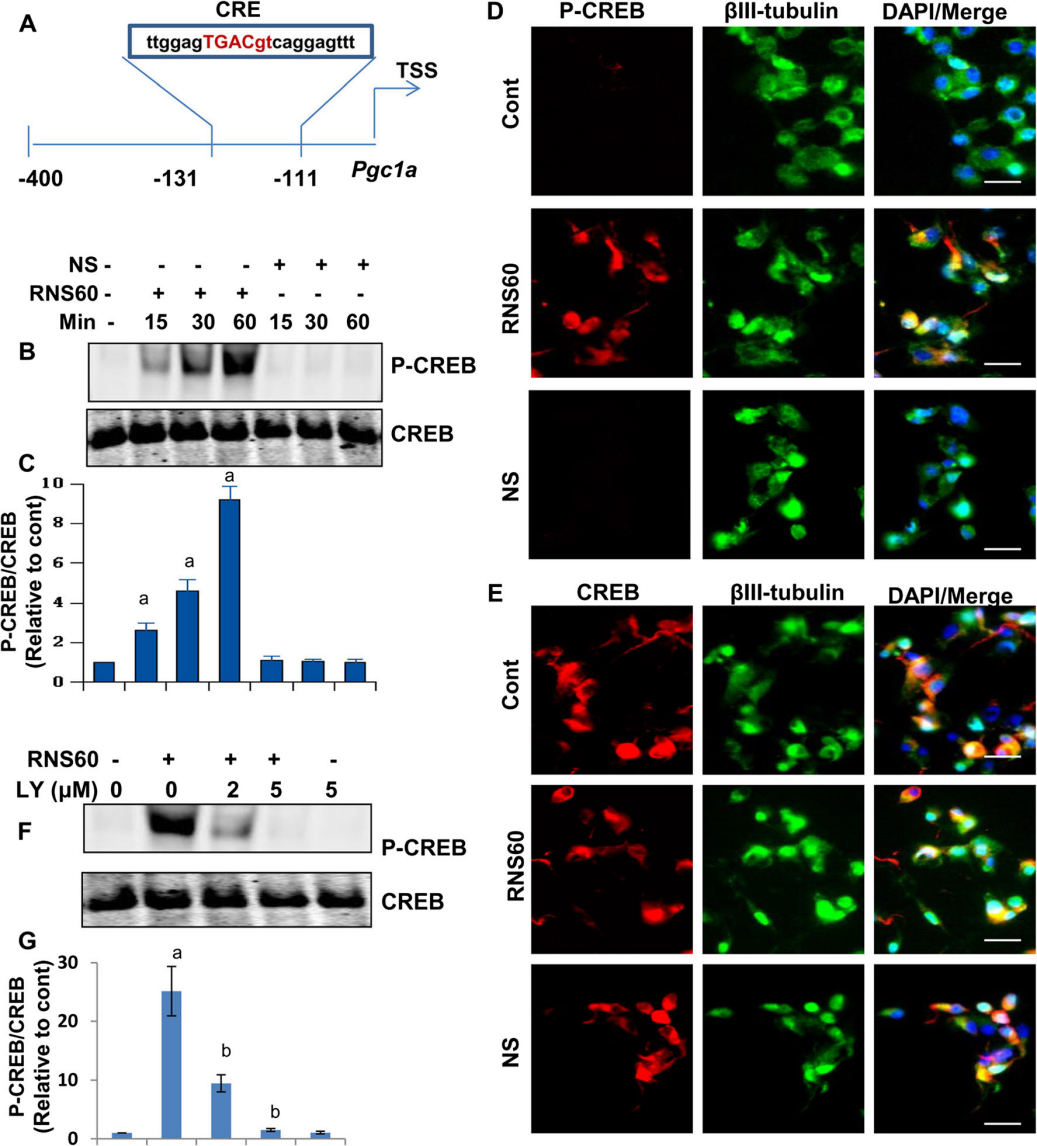
**Activation of CREB in MN9D mouse neuronal cells by RNS60 via PI3K**. **a** *Pgc1α* gene promoter analysis shows the presence of a consensus cAMP response element (CRE) near the transcription start site (TSS). **b** Cells were treated with 10% *v/v* RNS60 or NS for different minute intervals under serum-free condition followed by monitoring the levels of phospho (P)-CREB and total (t) CREB by Western blot. **c**) Bands were scanned and values of (P-CREB/tCREB) are expressed as relative to control. Results are mean ± SD of three different experiments. ^*a*^ *p* < 0.001 versus control. After 60 min of treatment, cells were double-labeled with either P-CREB & β-tubulin (**d**) or P-CREB & β-tubulin (**e**). Results represent three independent experiments. **f** Cells treated with different concentrations of LY294002 for 30 min were stimulated with 10% *v/v* RNS60 for 60 min followed by monitoring the levels of P-CREB and tCREB by Western blot. **g** Bands were scanned and values of (P-CREB/tCREB) are expressed as relative to control. Results are mean ± SD of three different experiments. ^*a*^ *p* < 0.001 versus control; ^*b*^ *p* < 0.001 versus RNS60

Next, we investigated how PI3K coupled the transcription of *Pgc1a*. While analyzing the *Pgc1a* gene promoter by MatInspector V2.2 search machinery, we found a consensus cAMP response element (CRE) very close to the transcription start site (Fig. 9a). Since CREB can bind to CRE and we have previously found the involvement of class IA PI3K in the activation of CREB (Khasnavis et al. 2012), we examined if PI3K coupled the transcription of *Pgc1a* via activation of CREB in RNS60-treated neuronal cells. At first, we monitored if RNS60 could induce the activation of CREB in MN9D neuronal cells. It is clearly evident from P-CREB and total CREB Western blots that RNS60, but not NS, markedly induced the phosphorylation of CREB within minute intervals without modulating the level of total CREB (Fig. 9b). These results were further confirmed by P-CREB (Fig. 9d) and total CREB (Fig. 9e) immunostaining of RNS60- and NS-treated cells. Furthermore, consistent to our earlier finding in microglial cells (Khasnavis et al. 2012), here we have also observed that RNS60 induces the activation of CREB in MN9D neuronal cells via PI3K as RNS60-mediated phosphorylation of CREB was dose-dependently inhibited by LY294002 (Fig. 9f–g).

**Fig. 10:**
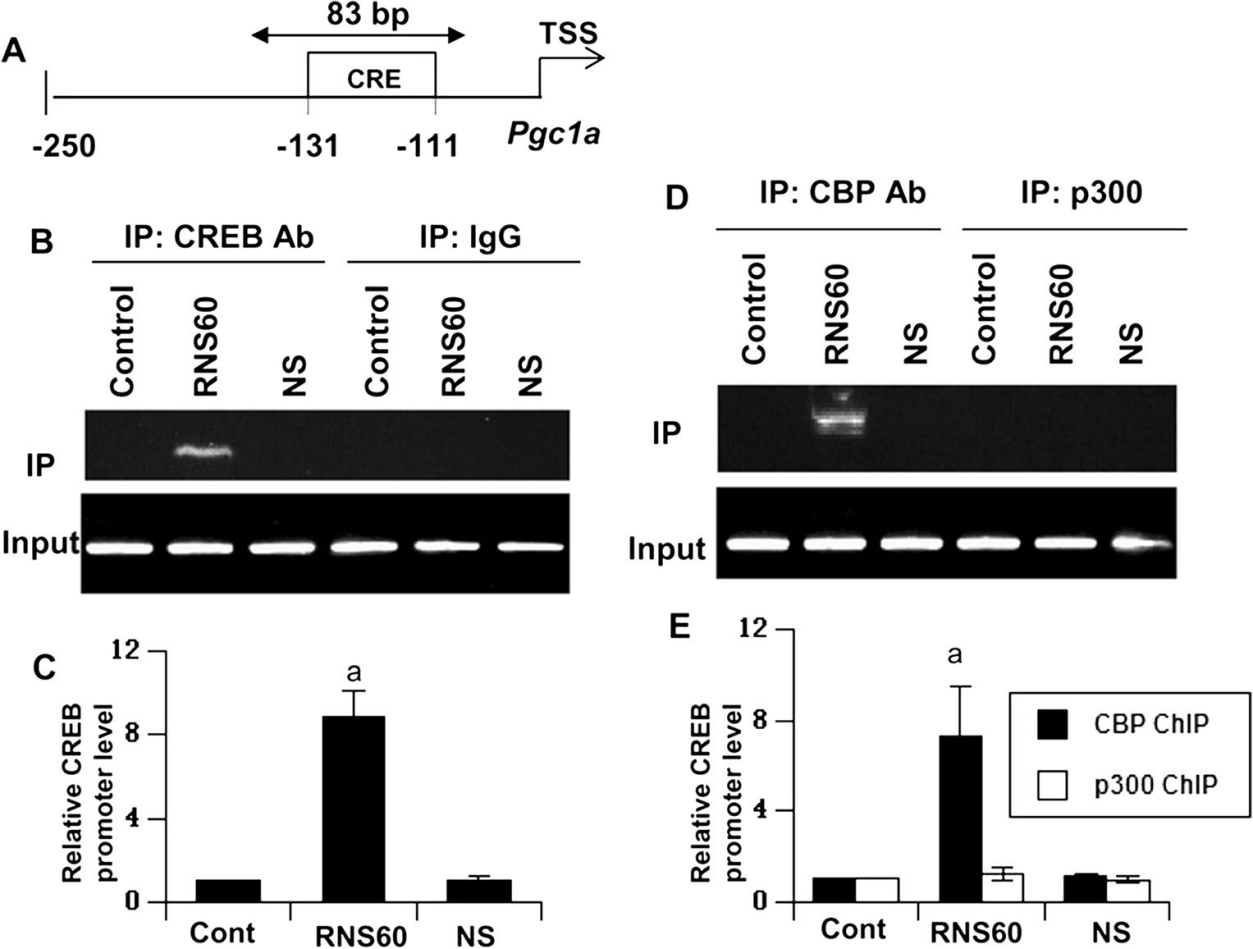
**RNS60 induces the recruitment of CREB to the** ***Pgc1a*** **promoter in MN9D neuronal cells**. **a** Position of the *Pgc1a* promoter that was targeted during ChIP analysis. Cells were treated with 10% *v/v* RNS60 or NS for 2 h and the recruitment of CREB to the *Pgc1α* promoter was monitored by ChIP followed by PCR (**b**) and real-time PCR (**c**). Values (CREB Ab IP/IgG IP) are expressed as relative to control. Results are mean ± SD of three different experiments. ^*a*^ *p* < 0.001 versus control. The recruitment of CBP and p300 to the *Pgc1α* promoter was monitored by ChIP followed by PCR (**d**) and real-time PCR (**e**). Values (CBP or p300 Ab IP/IgG IP) are expressed as relative to control. Results are mean ± SD of three different experiments. ^*a*^ *p* < 0.001 versus control

**Fig. 11:**
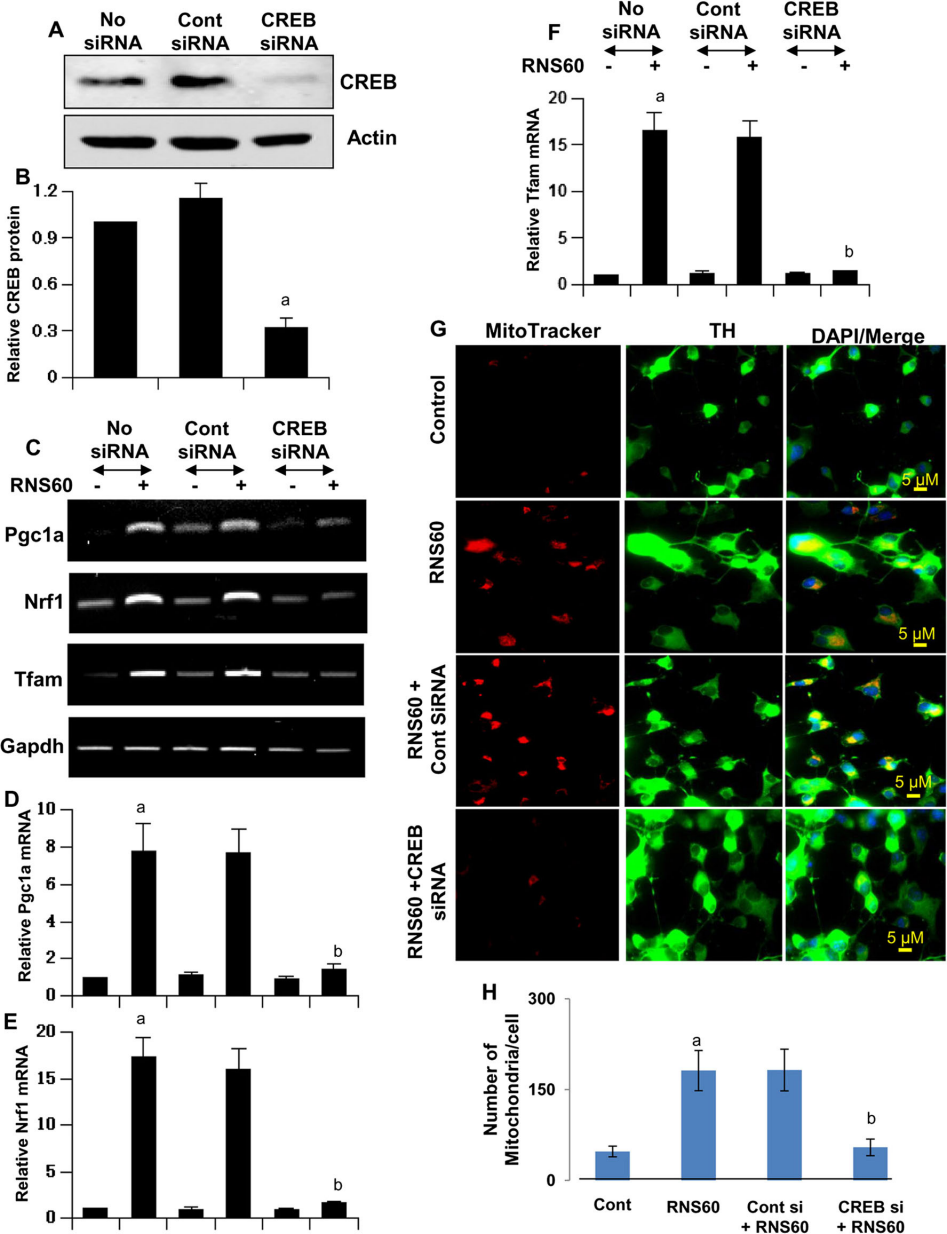
**SiRNA knockdown of CREB suppresses RNS60-mediated increase in PGC1α and upregulation of mitochondrial biogenesis in MN9D neuronal cells**. Cells were transfected with control or CREB siRNAs. After 48 h of transfection, the level of CREB was monitored by Western blot (**a**). Actin was run as loading control. Bands were scanned and values (CREB/Actin) presented as relative to control (**b**). Results are mean ± SD of three different experiments. ^*a*^ *p* < 0.001 versus control siRNA. After 48 h of siRNA transfection, cells were treated with 10% *v/v* RNS60 for 2 h followed by monitoring the mRNA expression of *Pgc1α*, *Nrf1* and *Tfam* by semi-quantitative RT-PCR (**c**) and real-time PCR (**d**, *Pgc1a*; **e**, *Nrf1*; **f**, *Tfam*). Results are mean ± SD of three different experiments. ^*a*^ *p* < 0.001 versus control; ^*b*^ *p* < 0.001 versus control siRNA-RNS60. **g**) After 48 h of transfection, cells were treated with 10% *v/v* RNS60 for 24 h followed by double-labeling for MitoTracker and TH. **h**) Number of mitochondria was counted and expressed as mitochondria per cell. Data are mean ± S.D. of three independent experiments. ^*a*^ *p* < 0.001 versus control; ^*b*^ *p* < 0.001 versus control siRNA-RNS60

Next, by using ChIP analysis, we examined whether CREB was in fact recruited to the *Pgc1a* promoter in MN9D neuronal cells after RNS60 treatment. Upon immunoprecipitation of chromatin fragments by antibodies against CREB, we were able to amplify 83-bp fragments flanking the CRE (Fig. 10a) in RNS60-treated, but neither NS-treated nor control untreated, neuronal cells (Fig. 10b–c). On the other hand, no amplification product was observed in any of the immunoprecipitates obtained with control IgG (Fig. 10b–c), suggesting the specificity of these interactions. Furthermore, immunoprecipitation of chromatin fragments by antibodies against CBP and p300 suggests that RNS60 induced the recruitment of CBP, but not p300, to the *Pgc1a* gene promoter in MN9D neuronal cells (Fig. 10d–e). Consistent to the recruitment of CREB to the *Pgc1a* promoter, siRNA knockdown of CREB (Fig. 11a–b) suppressed RNS60-mediated upregulation of *Pgc1a* mRNA (Fig. 11c–d) in MN9D neuronal cells. Accordingly, siRNA knockdown of CREB abrogated RNS60-mediated upregulation of Nrf1 (Fig. 11e) and Tfam (Fig. 11f) and increase in mitochondrial biogenesis (Fig. 11g–h). Together, these results demonstrate that RNS60 increases mitochondrial biogenesis in neuronal cells via class IA PI3K-CREB-mediated upregulation of PGC1α.

## Discussion

### RNS60 Stimulates Mitochondrial Biogenesis in Dopaminergic Neurons

The mitochondria is the most important organelle in cells that not only participates in energy homeostasis and metabolism but also play significant roles in other biological processes like signal transduction, aging, oxidative stress, and apoptosis (Mizuno et al. 1995; Baloyannis 2006; Zsurka and Kunz 2015). Accordingly, the abundance, morphology, and functional properties of mitochondria are finely tuned to meet cell-specific energetic, metabolic, and signaling demands. As a result, decreased mitochondrial biogenesis and function are seen in many pathological conditions including neurodegenerative disorders (Dong et al. 2015; Wang et al. 2016). Therefore, the ability to manipulate or influence mitochondrial biogenesis in neurons may be of therapeutic importance for neurodegenerative diseases. Although there may be other approaches to stimulate mitochondrial biogenesis, here we introduce a simple saline-based agent to achieve this. RNS60 is an electrokinetically modified saline that contains charge-stabilized nanobubbles, but no active pharmaceutical ingredients. Due to Taylor-Couette-Poiseuille (TCP) turbulence, RNS60 is proposed to contain charge-stabilized nanostructures consisting of an oxygen nanobubble core surrounded by an electrical double-layer at the liquid/gas interface (Khasnavis et al. 2012). Several lines of evidence presented in this manuscript clearly demonstrate that RNS60 is capable of upregulating mitochondrial biogenesis in dopaminergic neurons. Our conclusion is based on the following findings. *First,* we observed that RNS60, but not NS, RNA10.3 or PNS60, stimulated mitochondrial biogenesis in MN9D dopaminergic neuronal cells. Interestingly, RNS60 did not increase lysosomal biogenesis in MN9D cells. *Second,* RNS60, but not NS, RNS10.3 or PNS60, upregulated the expression of *Nrf1*, *Tfam*, *Mcu*, and *Tom20*, genes associated with mitochondrial biogenesis, in MN9D cells. However, RNS60 did not modulate the expression of *Tfeb* and *Lamp2*, genes associated with lysosomal biogenesis. *Third,* electron microscopy also revealed increase in mitochondria, but not lysosomes, by RNS60 in MN9D cells. *Fourth,* RNS60 also increased mitochondrial biogenesis in primary mouse dopaminergic neurons. *Fourth,* while the level of TFAM decreased in the nigra of MPTP mouse model of PD, RNS60 treatment upregulated and/or protected nigral TFAM from MPTP toxicity. Recently we have delineated protection of dopaminergic neurons and restoration of locomotor activities in MPTP-intoxicated mice by RNS60 (Khasnavis et al. 2014). Since mitochondrial dysfunction is known to contribute to nigrostriatal pathology in PD patients and in animal models of PD (Mizuno et al. 1995; Surmeier and Sulzer 2013), our current results suggest that this mitochondria-boosting effect of RNS60 may play a role in RNS60-mediated protection of the nigrostriatum in MPTP mouse model.

### RNS60 Requires PGC1α to Stimulate Mitochondrial Biogenesis

Mitochondrial biogenesis is a complex process that requires a coordinated regulation of synthesis, import, and incorporation of proteins and lipids to the existing mitochondrial reticulum, as well as replication of the mitochondrial DNA. A major breakthrough in our understanding of how mitochondrial biogenesis is coordinately regulated was the identification of NRF-1, NRF-2 and PPARγ, transcription factors that are responsible for mitochondrial biogenesis. Accordingly, it has been found that PPARγ coactivator 1α (PGC1α), functioning as a coactivator of NRF-1, NRF-2 and PPARγ, is capable of integrating various physiological signals and enhancing mitochondrial biogenesis (Wu and Boss 2007; Wareski et al. 2009; Villena 2015). Accordingly, we observed upregulation of PGC1α by RNS60 in MN9D neuronal cells. Peripheral administration of RNS60 also increased PGC1α in THir neurons as well as other brain cells in vivo in the nigra of MPTP intoxicated mice. Suppression of *Nrf1*, *Tfam*, *Mcu*, and *Tom20* gene expression and attenuation of mitochondrial biogenesis in RNS60-stimulated neuronal cells by siRNA knockdown of PGC1α suggests that RNS60 stimulates mitochondrial biogenesis via PGC1α.

### RNS60 Upregulates PGC1α in Neuronal Cells via Class IA PI3K-Mediated Activation of CREB

Mechanisms by which PGC1α is upregulated are poorly understood. Recently we have delineated that RNS60 increases IκBα in microglia via the activation of class IA PI3K p110α/β, but not class IB PI3K p110γ (Khasnavis et al. 2012). PI3K is a key signaling molecule implicated in the regulation of a broad array of biological responses including metabolism, cell growth, cell survival, and apoptosis (Koyasu 2003). In case of class IA PI3K, the p85 regulatory subunit interacts with the IRS-1 through its SH2 domain and thus recruits the p110 catalytic subunit (p110α/β) to the cell membrane, resulting in the activation of downstream signaling molecules like Akt/protein kinase B and p70 ribosomal S6 kinase (Koyasu 2003). While, for class IB PI3K, p110γ is activated by the engagement of G-protein coupled receptors. The p110γ then catalyzes the release of phosphatidylinositol (3,4,5)-triphosphate as the second messenger from phosphatidylinositol (4,5)-bisphosphate and activates downstream signaling molecules (Franke et al. 1997). Here we demonstrate that RNS60 induces the activation of both the subunits of class IA PI3K (p110α and p110β) without modulating class IB PI3K p110γ in MN9D neurons, suggesting the specific activation of class IA p110α/β PI3K in dopaminergic neuronal cells. Earlier by using Atomic Force Microscopy, we have demonstrated that RNS60 contains nanobubbles and that nanobubble composition of RNS60 is different from other control saline solutions (Khasnavis et al. 2012). Nanobubbles may interact with cells based upon their charge and size as these charged entities possess the necessary energetics to affect the membrane. Consistent to the presence of activated PI3K at the membrane, RNS60 containing charge-stabilized nanobubbles induced the recruitment of PI3K subunits to the membrane of neuronal cells. Abrogation of RNS60-mediated increase in PGC1α and upregulation of mitochondrial biogenesis in neuronal cells by inhibitors of PI3K suggest the involvement of PI3K in RNS60-mediated increase in mitochondrial biogenesis. Furthermore, we have seen that a consensus CRE is present in the *Pgc1a* promoter near the transcription start site and that RNS60 induced the recruitment of CREB and CBP to this CRE in *Pgc1a* promoter. Suppression of RNS60-induced CREB activation by inhibition of PI3K suggests that RNS60 induces the activation of CREB in neuronal cells via PI3K. Recently, we have delineated that Akt, the downstream mediator of PI3K, is responsible for the activation of CREB in RNS60-treated microglial cells (Khasnavis et al. 2012). Therefore, it is possible that a similar pathway exists in dopaminergic neurons as well. Furthermore, attenuation of RNS60-mediated increase in PGC1α and mitochondrial biogenesis by siRNA knockdown of CREB suggests that RNS60 increases the transcription of *Pgc1a* in neuronal cells via class IA PI3K-mediated activation of CREB, resulting in increased mitochondrial biogenesis.

In summary, we have demonstrated that RNS60 upregulates mitochondrial biogenesis in neuronal cells via class IA PI3K-CREB-mediated upregulation of PGC1α. These results highlight a novel mitochondria boosting property of RNS60 and suggest that this simple modified saline may be explored to rejuvenate degenerating neurons in PD and other neurodegenerative disorders where impaired mitochondrial function serves as a causative factor.
